# How Do Theories of Cognition and Consciousness in Ancient Indian Thought Systems Relate to Current Western Theorizing and Research?

**DOI:** 10.3389/fpsyg.2016.00343

**Published:** 2016-03-15

**Authors:** Peter Sedlmeier, Kunchapudi Srinivas

**Affiliations:** ^1^Institut für Psychologie, Technische Universität ChemnitzChemnitz, Germany; ^2^Department of Philosophy, Pondicherry UniversityPuducherry, India

**Keywords:** consciousness, Samkhya-Yoga, cognition, meditation research, extraordinary cognition

## Abstract

Unknown to most Western psychologists, ancient Indian scriptures contain very rich, empirically derived psychological theories that are, however, intertwined with religious and philosophical content. This article represents our attempt to extract the psychological theory of cognition and consciousness from a prominent ancient Indian thought system: Samkhya-Yoga. We derive rather broad hypotheses from this approach that may complement and extend Western mainstream theorizing. These hypotheses address an ancient personality theory, the effects of practicing the applied part of Samkhya-Yoga on normal and extraordinary cognition, as well as different ways of perceiving reality. We summarize empirical evidence collected (mostly without reference to the Indian thought system) in diverse fields of research that allows for making judgments about the hypotheses, and suggest more specific hypotheses to be examined in future research. We conclude that the existing evidence for the (broad) hypotheses is substantial but that there are still considerable gaps in theory and research to be filled. Theories of cognition contained in the ancient Indian systems have the potential to modify and complement existing Western mainstream accounts of cognition. In particular, they might serve as a basis for arriving at more comprehensive theories for several research areas that, so far, lack strong theoretical grounding, such as meditation research or research on aspects of consciousness.

Almost unknown to Western academic psychologists, there is a huge collection of ancient Indian thought systems that attempt to describe and explain human experience and behavior. By thought systems we mean interpretations of the famous Vedas or reactions to these ancient Indian scriptures that encompass psychology, religion, and philosophy. We will refer to these hereafter simply as systems. Some applied parts of the respective writings are sometimes referred to in Western therapeutic approaches that deal with meditation (e.g., Kabat-Zinn, 1996; Hayes et al., 1999; Wallace and Shapiro, 2006; Walsh and Shapiro, 2006; Linehan, 2007). However, the theories contained in the Indian systems were not developed to describe (psychological) clinical or therapeutic processes—in fact, they did not include any clinical aspects at all (with the exception of Ayurveda) although they propose methods to improve life and gain wisdom (e.g., Feuerstein, 2001). These systems are basically theories that cover in principle all the main aspects of psychology, such as cognition, behavior, personality, emotion, and volition. One reason why these systems have never made it into Western textbooks might be that they are not purely psychological: They also contain elements of religion associated with Buddhist and Hindu practice (e.g., mentioning of personal gods) and of philosophy.

In this article we will try to lay out the psychological parts of two prominent Hindu systems, Samkhya and Yoga, usually treated as a single system. We put our focus on what this system has to say about cognition (including consciousness). It will be seen, however, that cognition cannot be fully isolated from the other aspects of human experience and behavior, as has usually been attempted in mainstream Western psychology. Moreover, it will become clear that Indian systems of thought contain applied parts that give directions for how to lead a fulfilled life, an aspect that has only recently found increased interest in Western psychology.

We are, of course, not the first ones attempting to connect Eastern and Western theoretical views. Several authors have already introduced Buddhist approaches as a theoretical basis for research on meditation. For instance, Lutz et al. (2007) describe in detail a Tibetan Buddhist approach to meditation, and Grabovac et al. (2011) explain a more recent Burmese Theravada Buddhist theoretical approach. There are also a few instances of specific topics, for which a connection between Indian systems and Western theorizing has been drawn, such as the issue of consciousness and deep sleep examined by Thompson (2015). The basic difference between our attempt and previous approaches is that we do not begin with a specific topic (e.g., meditation or deep sleep) and see what Eastern approaches have to say about it but instead, we start with a complete Indian system and explore in which areas in Western psychology general hypotheses derived from this system could make a difference. Here, for want of space, we will concentrate on a prominent combined Indian system: Samkhya-Yoga. Although we are not aware of any systematic research in the West that explicitly relates to the (rather broad) hypotheses to be derived from the Samkhya-Yoga system, some Western researchers might have had such hypotheses in mind when conducting their research. There are, however, plenty of studies whose results can be used to evaluate the Samkya-Yoga view and we will summarize and evaluate this evidence below. In addition, for each hypothesis, we will make suggestions for more precise hypotheses to be examined in further research.

Before we begin, it is probably necessary to clarify two central issues. One is the apparent incompatibility of the religious aspects contained in the Indian systems on the one hand and secular Western (psychological) science on the other: Is it really possible to build a bridge between the two? And the other question that arises if one comes to a positive conclusion for the first issue is this: How can such a bridge be built, with such different contexts and nomenclatures?

If one regards the Indian systems as solely religions that tell one what and what not to believe, then such an endeavor would make little sense. However, if one sees the psychological content in these systems as empirically grounded theories, then there is nothing that in principle speaks against making such a comparison. Indeed, Buddhist insights, for instance, rest on the experiences the Buddha (and also many of his followers) obtained in “trial-and-error” experiments (Jayatilleke, 1963, p. 464). One might argue that for the Hindu systems, the situation is different because many holy texts are claimed to have been revealed and carry with them the connotation of “truth” in an unquestionable sense (see Klostermaier, 2006, p. 11). But here one could also advance the argument that the psychological insights reported in Hindu texts are very likely based on personal experiences—at least, this is our working hypothesis. Support for this empirical and investigative view comes from contemporary scholars (e.g., Phillips, 2009, p. 39) and leading Hindu figures of the recent past. For instance, Swami Vivekananda was of the opinion that “if a religion is destroyed by such investigations, it was then all the time useless, unworthy superstition; and the sooner it goes the better” (Swami Jitatmananda, 2004, p. 171). And Sri Aurobindo who kept an elaborate diary on the effects of his yoga practice over many years (Sri Aurobindo, 2001) came to the conclusion that yoga, the applied part of Hindu theory, “is nothing but practical psychology” (Sri Aurobindo, 1996, p. 39). Although the original evidence is more akin to qualitative data gathered in single-case designs in contemporary psychology, also sometimes termed *phenomenological* evidence (e.g., Pekala, 1987), it is, in our view nonetheless as empirical as can be (see also Gallagher and Zahavi, 2013). One might, of course, still question the sources of the respective theories but even for Western mainstream theories it is often not clear how they originated. Anyway, the scientific method (e.g., Bunge and Ardila, 1987) is concerned not so much with the way theories are “found” but with how they can be tested, which makes them acceptable for science or not. If the scientific method can be applied to a theory (see Sedlmeier, 2014, for arguments why this is the case for Indian psychology), and if that theory is wrong, it will eventually be found out. So to summarize our conclusion for the first potentially critical issue, what will be compared in this article is (ancient Indian) psychology with (contemporary Western) psychology, and *not* religion (or philosophy) on the one hand with psychology on the other.

The second potentially problematic issue arises from the way ancient Indian psychology is presented. Because it is embedded in a religious or philosophical context, and, of course, because it was compiled a long time ago, the language and arguments used are not familiar to present-day psychologists. An additional problem arises because the original texts were written in ancient languages such as Sanskrit and Pali, and translations are often ambiguous; that is, different scholars may translate (and interpret) the same expressions differently. So what scholars who deal with these systems (mostly philosophers and philologists by profession) usually do is to add the original terms when they use the English translations. We will also do this occasionally for very central terms but to minimize the negative impact on the readability of the paper, we will use mostly English translations and provide a glossary of the corresponding Sanskrit terms (without the potentially confusing diacritical marks) in the Supplementary Material. Please note that we will not attempt to do yet another of the already innumerable exegeses of the original texts, and we also do not strive for a comprehensive coverage of all the diverging interpretations of these texts. What we want to convey, however, are all aspects that are central to the theories of cognition contained in the Indian views in a way most experts in the respective fields would agree upon.

Despite the two above-mentioned potential obstacles, we think that the task attempted here is overdue and worthwhile, because if the discrepant theoretical aspects contained in ancient Indian systems eventually turn out to be strongly empirically corroborated, they hold a large potential to enrich psychological theorizing by giving a comprehensive theoretical framework for some hitherto more or less unrelated areas of research and by introducing new hypotheses into mainstream psychology. Because our argument is of a general nature, we will be concerned not so much with minute details of the system we describe but more with its central aspects, and especially those that are not or only partly consistent with Western views. To explicate these aspects we thought it necessary to also give some background information.

## Cognition in Samkhya-Yoga

Although occasionally objections can be found to such a view (e.g., Ranganathan, 2008), the two systems of Samkhya and Yoga are usually seen as strongly related and therefore often treated together, which we also will do here. The system of Samkhya is ascribed to one mythical Kapila, but the earliest works that date back to the second century BCE seem to have been lost and the *Samkhya Karika* of Ishvaraskrishna (about 200 CE, e.g., Dasgupta, 1997, p. 212) is supposed to be the authentic text that brings out the essence of Samkhya philosophy (Raju, 1985, p. 305). Yoga represents the practical aspect of Samkhya. Therefore, the two are treated as allied systems. The main source of Yoga philosophy is Patañjali's *Yogasutras* (see Whiteman, 1993; Woods, 1998, for translations). It is not clear when, exactly, the Yogasutras were written, but very probably it was somewhere between the second century BCE (not earlier than 147 BCE, according to Dasgupta, 1997, p. 212) to the fifth century CE (Flood, 2004).

We decided on Samkhya-Yoga because apart from containing quite elaborate psychological theories, it is also arguably one of the most important of the Indian systems, and because it contains theoretical aspects that go beyond Western psychology to a greater extent than other Indian systems^1^. The importance of Samkhya-Yoga can be seen in that references to it are to be found everywhere in the most important texts, such as the early *Upanishads*, the *Bhagavad-Gita*, and also other parts of the *Mahabharata* (Puligandla, 1997, p. 119). Samkhya-Yoga directly relates to the old Indian scriptures summarized under the name *Veda* (wisdom), which date back several millennia^2^. It is, however, difficult to derive psychological theories directly from the Vedas because they contain considerable portions that are concerned with rituals and are often written in a poetic and aphoristic style. Therefore, we relied on a systematically elaborated system that contains widely accepted scholarly explanations of the Vedas^3^.

### Cognition in everyday life

In Yoga and Samkhya, a person consists of two “components,” one material and one not (for details see Dasgupta, 1930; Chatterjee and Datta, 1984; Puligandla, 1997; Jha, 2008; Rao and Paranjpe, 2008). The material one, *prakriti* (roughly meaning nature), is composed of three “qualities,” the three *gunas*: *sattva* (purity), *rajas* (energy), and *tamas* (inertia). Prakriti, in the form the three gunas, is the essence of the universe as well as the basis for the personality of a given person. The specific personality, as well as everything material (including the mind), is the product of a “mixture” of the three gunas.

It is seen as beneficial if sattva is strong because people with a high level of sattva are expected to have a positive view of the world, a well-meaning attitude toward others, to be disciplined, calm, and relaxed, and to have a high stress tolerance and a healthy lifestyle. People with a high level of rajas, in contrast, have difficulties relaxing and prefer actions that bring them short-term pleasure or relief but may be harmful in the long run. Moreover, they tend to have an unhealthy lifestyle and tend to waste their energy. Finally, people with a high level of tamas are dissatisfied with their lives and are most likely to neglect their health. However, the mixture of the three energies is not seen as necessarily stable: Increasing the level of sattva is generally seen as a desirable goal. And only if sattva is dominant in a person will this person be able to achieve extraordinary spiritual aims (see below).

**Figure 1:**
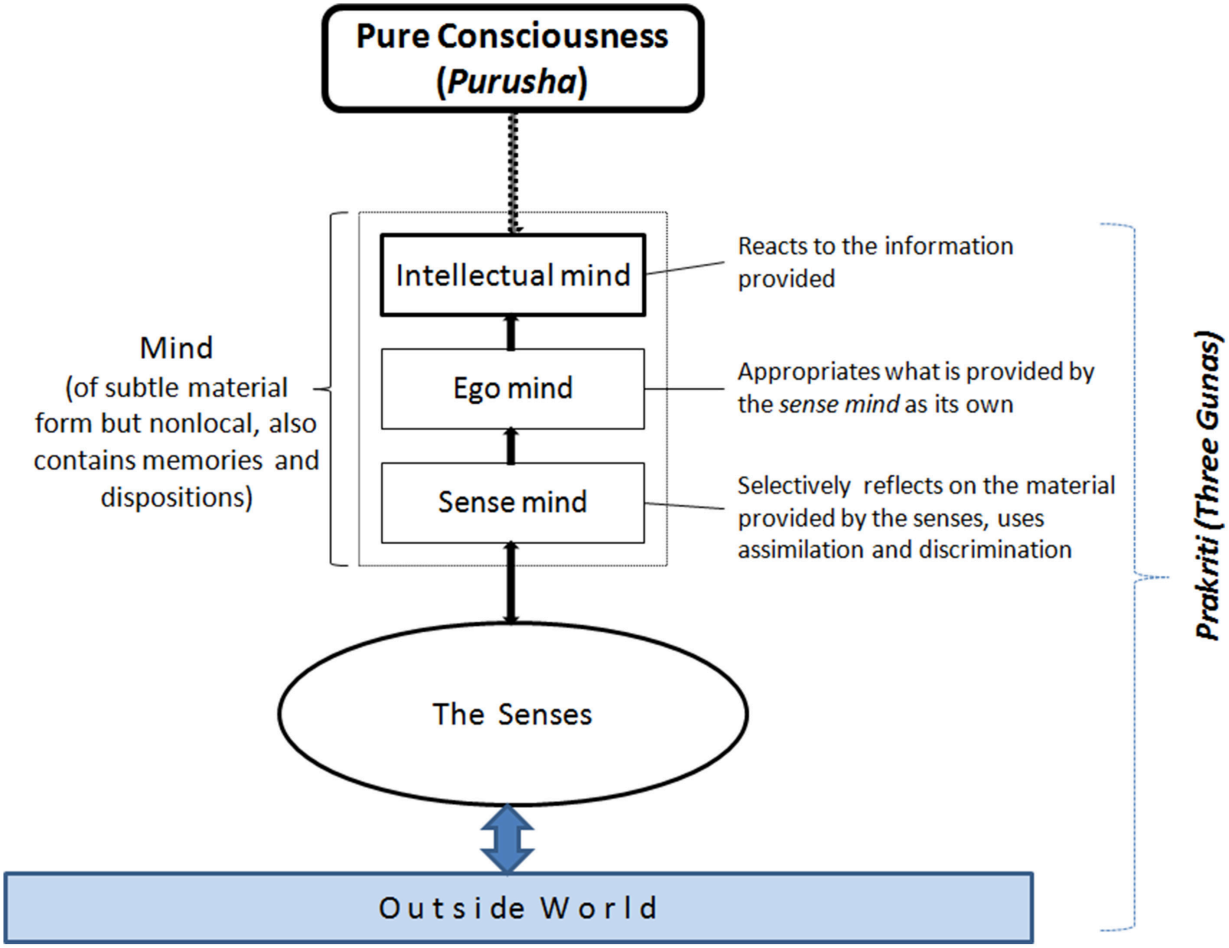
**Graphic description of how cognition works according to Samkhya-Yoga**.

The nonmaterial component of a person (and of the world) is *purusha*, sometimes translated as *true person* or *true self*, but, especially by Indian academic writers, often rendered as *pure consciousness*, the term that we also use here. Note that whereas the concept of consciousness as used in Western thinking is always intentional, that is, of or about something, pure consciousness has no qualities or characteristics of its own but underlies all our being and knowing. Together, prakriti and purusha constitute the manifest world as well as the person in its full sense. Figure 1 indicates the relationship between purusha and prakriti (everything except purusha) and illustrates how a person cognizes according to the Yoga system. The mind contains dispositions and memories of all sorts and has three functional aspects, sense mind, ego mind, and intellectual mind or intellect.

How, for instance, does perception work in this approach? The sense mind chooses an external object through the peripheral sense organs. This object is then appropriated by the ego mind that relates the object to itself (“*I* perceive the object”). Then, the intellect represents the object (takes on the form of the object) and makes a judgment or decides about possible actions. The intellect, which is by nature unconscious (part of *prakriti*), becomes conscious (and cognitions become possible) only when it makes contact with *purusha*. In other words, without the working of purusha it would not be possible to cognize at all. However, for untrained persons, the contact between purusha and the intellect only works in one direction. Whereas purusha “uses” the intellect, the intellect only reflects pure consciousness without “seeing” it in its pure form. However, this reflection gives us self-consciousness and our subjective experience of the world. The information the mind uses does not have to come from the senses alone. It might also come from inferences, verbal sources, or memory. In addition, it contains instinctual tendencies inherited from the effects of past actions and from previous lives that have their effect in the present life. The intellect itself contains memory contents form prior experiences (and from prior lives).

Let us illustrate the working of the mind with the help of an example. If, for instance, the senses are presented a banana, the sense mind gets all the impressions of color, shape, taste, and so forth, combines them, and separates the total unified object built in this way from other objects. Then the ego mind appropriates the object as its own, as in “*I* see a banana.” At this stage of perception, the object in question (e.g., a banana) is an object of one's experience but is not seen as an object of the objective world. The intellect makes it such an object through an assertion or a decision yielding something like “this is a banana” as the result of the decision process. Moreover, in conjunction with the other parts of the mind it collects different aspects of the banana, such as the banana tree, my eating it, its price, its nutrients, and so forth, and relates them to the perceived banana. This experience is also saved as a memory. However, the whole process only works with the help of pure consciousness that is “reflected” in the mind and senses.

All levels of the mind are assumed to have material form but they differ in subtleness, the intellect being the most subtle and the sense mind the least^4^. All components, however, are assumed to be of a vastly more subtle material than the body (including the brain) and the senses. Note that in Samkhya-Yoga, the mind, although material, is nonlocal; that is, it need not be situated in the brain (e.g., Rao, 2005). Most importantly, and of course fundamentally different from the Western view, the mind does not work on itself: Conscious cognition needs the connection between the intellect and the central component in the Yoga system: pure consciousness.

### Extraordinary cognition and how to obtain it

As already mentioned, under normal conditions, pure consciousness is only *reflected* in the intellect and not recognized. Gaining access to pure consciousness and dwelling therein is, in fact, the highest aim in the system of Yoga (to see reality as it is), to be brought about by making the sattva part of the three gunas dominant: One could even say that the goal of Yoga is the yogi's “sattvification” (“yogi” standing for the person pursuing the path of Yoga as described in the Yogasutras). The result of having gained access to pure consciousness and dwelling therein is known in the literature as, for instance, “enlightenment” or “realization.” And consequently, the Yoga system also has a procedure for reaching that desirable state.

How can one arrive at the state of pure consciousness? To understand this, one has to know that according to Yoga, the state of the conscious mind is constantly changing or fluctuating, and there are single units of awareness. These units can be valid cognitions arising from perception (as described above), inference, or verbal sources, but they can also be doubts and uncertain or false cognitions. Moreover, they can come from memory and they do not necessarily have to represent some real object or event. Sleep is also considered to be such a kind of unit. As long as there is this fluctuation of the mind, pure consciousness cannot be accessed; but it is already present and without its presence, the mind would not be able to produce conscious cognitions. Only if the fluctuations of the mind can be brought to a standstill will the connection between intellect and pure consciousness become strong enough to achieve the desired access and enable the yogi to dwell in pure consciousness^5^.

According to the Yoga philosophy, fluctuations of the mind are brought about by the existence of the so-called five *kleshas* or hindrances (see second book of Yogasutras). The suffering in life reflected in the fluctuations of the mind is due to ignorance about pure consciousness and therefore life becomes driven by sensory inputs and ego cravings. Not surprisingly, therefore, the first and fundamental hindrance is spiritual ignorance, that is, the inability to discriminate between good and evil, truth and falsehood, the permanent and the impermanent, and so forth. The other four hindrances are attachment to or involvement in the thirst, greed, and craving for enjoyment, hate or aversion of anything considered painful, the feeling of a (mistaken) personal identity, and the desire to continue to be what one is and the instinctive fear of death.

The way to attain freedom, that is, to attain access to pure consciousness, or realize one's true self is presented as a path involving eight stages that eventually help the mind reach quietness by completely restraining all its functions. This eightfold path consists of five steps that include rules of moral conduct as well as bodily and breathing exercises and the practice of focusing one's attention inward, and three steps that can be seen as concentration practices. In particular, the first five steps concern ethics, inner spiritual discipline, body postures, breath control, and control of the senses; and the last three steps consist of concentration, contemplation, and the meditative state of absorption. One might see the control of the senses as the bridge between the first four “outer stages” and the last three “inner stages,” whereas the last four parts (including control of the senses) can be regarded as aspects of meditation.

The control of the senses is achieved by different techniques that bring the mind's focus to a single point in the body and culminates in consciously withdrawing attention from anything that is distracting for the mind. Then, in the step of concentration, practitioners are to focus their mind on a chosen object without consciousness wavering from it. In this step, the meditators' (meta-) awareness is conscious of the act of meditating, of the object concentrated upon, and of their ego mind, that is, of themselves concentrating on the object. Then, in the stage of contemplation, consciousness of the act of meditation disappears. And in the state of absorption, the ego mind also dissolves, and meditators become one with the object.

On the way to attaining freedom, the yogi may (as in the Buddhist path) acquire several kinds of extraordinary cognitions (*siddhis*) when the last three steps mentioned above are applied in certain ways and to certain objects (see third book of the Yogasutras; see also Braud, 2008). Examples are knowledge of the past and future, clairvoyance, clairaudience, psychokinesis, and telepathy. But the ultimate aim of developing extraordinary cognition is (as in Buddhist approaches), liberation or enlightenment, which can be described in Samkhya-Yoga as gaining unobstructed access to and then dwelling in pure consciousness, without any further reincarnation.

## How may the Samkhya-Yoga view of cognition and consciousness complement and extend western theorizing?

Although there are many similarities between theories of cognition in contemporary Western psychology and the one outlined for Samkhya-Yoga, it should also have become clear that the theory of cognition described above *does* in several respects differ from the Western view, which is, of course, also not really uniform but contains many variations. We counted as “Western view” any theoretical approach within Western psychology we were aware of that had made it into a major journal.

In Samkhya-Yoga, there is a controlling instance, the mind, which governs how information is processed and acted upon. One might argue that the tri-partition into sense mind, ego mind, and intellect is different from Western conceptions of the mind but these conceptions seem not to have been specified in enough detail to allow for precise predictions that are different from those derivable from Western accounts.

Below, we briefly summarize several main points in which we think the views of cognition in Samkhya-Yoga complement and extend the Western views, in the form of four rather broad hypotheses. For each of the hypotheses, we first summarize empirical evidence that stems almost exclusively from research *not* concerned with theories from ancient Indian psychology, and then suggest ways to test the respective hypotheses in more specific ways.

### Three gunas hypothesis

According to the Samkhya system, every personality can be described as a mixture of three “qualities,” the three gunas: sattva, rajas, and tamas. Knowing the specific mixture for a given person allows for predictions about that person's experiences and behavior. This conception of personality is so different from prevalent Western personality theories that it warrants a hypothesis of its own.

#### Empirical evidence

The concept of the gunas has already been elaborated, mostly by Indian psychologists, and connected to issues of illness and psychological well-being (e.g., Lakshmi Bhai et al., 1975; Sharma, 1999; Narayanan and Krishnan, 2003; Sitamma, 2005; Murthy and Kumar, 2007; Rani and Rani, 2009; Suneetha and Srikrishna, 2009). There also have already been, mostly in the Indian context, several empirical investigations that focus on attempts to operationalize the guna concept with questionnaires (e.g., Mohan and Sandhu, 1986; Das, 1991; Marutham et al., 1998; Wolf, 1998; Stempel et al., 2006). Studies using these questionnaires generally found positive correlations between sattva guna and positive emotions and personality traits (e.g., Lakshmi Bhai et al., 1975; Sharma, 1999; Narayanan and Krishnan, 2003; Sitamma, 2005; Murthy and Kumar, 2007; Rani and Rani, 2009; Suneetha and Srikrishna, 2009). The results of the attempt to find factors that correspond with the gunas and the impact of several treatments on changes in the gunas were not in full agreement, but overall, they seem promising (for an overview, see Puta and Sedlmeier, 2014). Very recently, Puta (2016), in two large studies, developed a new comprehensive guna scale with nine separate subscales that each measure the gunas in one single behavioral category each (e.g., cognition, emotion, etc.). This scale does not suffer from the deficiencies that can be found in former attempts. In a further intervention study, she examined the effects of a yoga-based intervention program that included meditation, cognitive restructuring, managing energy and will-power, and mindful decision making and found substantial increases in sattva, as well as decreases in rajas and tamas.

#### Further research

Although this concept of personality might seem strange at first glance to many Western readers it is likely the result of long-term empirical investigations of differences in personality and could at least be regarded as a working model for further research, which could prove especially useful in examining the effects of meditation and other yoga practices. So far, in Western studies, with the exception of Puta's (2016) study, measurement to examine the effects of yoga and meditation (see next paragraph) has mainly relied on devices that were *not* derived from a theory of meditation. In contrast, sattvification is the main result to be expected from yoga practice and therefore, Samkhya-Yoga would suggest that a guna questionnaire be a central measurement device to assess the effects of yoga (including meditation). Moreover, it might be promising to explore whether predominant Western views, such as the Big-Five personality system (e.g., McCrae and John, 1992) could be complemented by this ancient view.

### Cognitive training hypothesis

The cognitive training hypothesis states that the practice of yoga has profound positive effects on all aspects of cognition understood in a very broad sense. This hypothesis is not as explicitly stated in the original writings as the other three dealt with in this paper, but it has received by far the highest attention in contemporary Western research. One could say that the main purpose of Samkhya-Yoga theory is to provide the basis for improving one's life and eventually discovering the “true reality” by acquiring extraordinary forms of cognition—that is, to “see” the world (including oneself) as it really is. But on the way toward fulfilling this goal, one should already expect positive changes in basically all psychological aspects that are open to improvement. Why? The applied part in the Yoga system, Patañjali's eightfold path prominently includes aspects of meditation. Practicing it, along with heeding ethical rules of conduct and performing bodily exercises, should lead to a person's sattvification accompanied by a reduction of the five hindrances [spiritual ignorance, greed, hatred, the feeling of a (mistaken) personal identity, and the desire to continue to be what one is]. As the main practice in yoga meditation is concentration, one should also expect increases in attention, concentration, and in general, cognitive abilities. Therefore, the practice of yoga should be expected to have quite comprehensive positive effects on all aspects of cognition, either directly or indirectly via modifications of emotions and personality traits.

We are not aware of any attempt to examine the combined effect of the eightfold path of the Yogasutras, or of comparative studies that, for instance, looked at the effect of following the ethics parts compared to the meditation part or a combination thereof. Moreover, terms are often used quite differently in Western contexts. Whereas the term *yoga* was originally meant to include all eight parts of the eightfold yoga path, in the West, it often stands for only one or two limbs of the path, that is, for the practice of body postures, sometimes combined with breath control exercises^6^. Also, the term meditation is usually used in a quite indiscriminative sense and many of the meditation techniques in use are recent elaborations of ancient techniques. But nonetheless, if beneficial effects can already be seen when the eightfold path is practiced to some degree, this should count as partial evidence for the more comprehensive hypothesis.

Although therapy was not an explicit concern in the original systems, in which adepts were usually required to come with a stable personality as a precondition to being accepted by a spiritual teacher (e.g., Feuerstein, 2001), there is a long tradition of incorporating techniques from Buddhist and Hindu yoga approaches, mostly some kind of meditation, into Western approaches to psychotherapy. Apart from these connections to clinical psychology, yoga techniques are only recently gaining more attention in mainstream academic psychology, but there is also another potential link: The cognitive training part of the Indian systems might be seen as having many similarities to recent developments in health psychology, positive psychology, and related fields (Gillham and Seligman, 1999; Seligman and Csikszentmihalyi, 2000; Kahneman et al., 2003; Seligman et al., 2005, 2006; Robbins, 2008; Ryff and Singer, 2008; Diener and Ryan, 2009; Diener, 2013; Lynn et al., 2015). In these recent Western approaches, a central aim is to explore ways to make people happier, also emphasizing eudaemonic, in contrast to hedonic, conceptions of happiness^7^. Among the techniques proposed there, one finds traditional relaxation methods but increasingly also yoga and meditation techniques, including forms of “meditation in movement” such as Qi Gong (for overview see Malaktaris et al., 2015).

There is a steadily growing number of studies examining the effects of yoga techniques, including meditation. Given the already huge number of such studies, especially those on the effects of some kind of meditation, and for want of space, we will restrict this overview to summary reports (mostly meta-analyses) of the *psychological* effects of yoga and meditation, separately for studies with clinical and nonclinical populations.

#### Psychological effects of yoga/meditation with clinical populations

Quite a number of studies have examined the effects of yoga and meditation on measures of mental health in clinical populations. In these studies, the term “yoga” has been used in many different variations, sometimes referring to only certain postures, sometimes to combinations of postures and breath exercises, and sometimes also including some kind of (concentrative) meditation; but in no case did we find effects of adhering to ethical or spiritual aspects explicitly mentioned.

Büssing et al. (2012a) give an overview of the summary studies on the clinical effects of yoga interventions on mental (and physical) health. Aspects of mental health dealt with in these studies were depression, fatigue, anxiety disorders, stress, and posttraumatic disorders. For all these aspects, beneficial effects of yoga had been found. However, the authors caution that many of the studies are of poor methodological quality, and that there is a huge heterogeneity in the populations studied, the yoga interventions, the comparison groups, and the outcome measures. The effects of yoga on fatigue were reported in more detail in a separate meta-analysis that included 19 studies with patients suffering from various illnesses (Boehm et al., 2012). Overall, the effect of yoga was rather small, amounting to *d* = 0.27 standard deviation units. In a further meta-analysis that concentrated on yoga's pain-related effects, apart from finding moderate effects for decreasing pain of different sorts, Büssing et al. (2012b) reported a positive average effect (in standard deviation units) on pain patients' mood stages of *d* = 0.65, in six yoga studies. Cramer et al. (2013) summarized the outcomes of yoga in nine studies with patients with depressive disorders and individuals with elevated levels of depression. They found moderate evidence (*d* = 0.69) for a short-term effect of yoga on severity of depression compared to usual care. The evidence was more limited when effects of yoga were compared to relaxation training or aerobic exercises. A subgroup analysis indicated that yoga was more effective if body postures were combined with meditation.

Whereas studies on the effects of yoga (understood in the more narrow sense of body postures and breath exercises, sometimes combined with meditation) on mental health seem to have been conducted only over the past four decades (Büssing et al., 2012a), there have long been attempts to bring together meditation and psychotherapy. In an early literature survey, Smith (1975) acknowledged therapeutic benefits of meditation but concluded that the evidence available was insufficient to decide whether meditation or just context or expectation effects caused the positive outcomes. A more recent narrative review by McGee (2008) summarized beneficial effects on a wide variety of mental health problems and in further meta-analyses, positive effects on substance abuse (Alexander et al., 1994b) and on general health (Grossman et al., 2004) were found. However, another comprehensive review concluded that overall, “the therapeutic effects of meditation practices cannot be established based on the current literature” (Ospina et al., 2007, p. 6). Similarly, a more recent meta-analysis by Goyal et al. (2014) found moderate effects (*d* = 0.23 to 0.38) of meditation on anxiety, depression, and pain but low evidence for stress and mental-health-related quality of life. These authors also found no evidence that meditation programs were noticeably better than active treatment (for similar results see Strauss et al., 2014).

Despite a general impression of (moderately) positive effects, many studies are still plagued with methodological problems and problems of interpretation because of the substantial heterogeneity of studies in almost all imaginable aspects (see also Forfylow, 2011). Moreover, it is still unclear whether yoga and meditation are substantially better than existing therapies (e.g., Smith et al., 2007). However, taken together, the evidence suggests that yoga and meditation can help reduce a variety of mental problems such as anxiety, depression, and pain at least in some clinical populations and might, if not replace existing therapies, at least serve as an adjunct treatment.

#### Effects of yoga/meditation in nonclinical populations

As already mentioned, the yoga path was originally not meant to solve mental or physical problems but as a way to gain enlightenment. Therefore, nonclinical populations would be more suitable to test the predictions of Samkhya-Yoga, but such populations have seldom been examined, at least not when studying the effects of body-centered yoga techniques. The only meta-analysis we could find was one by Patel et al. (2012) that examined the effects of a variety of yoga techniques on physical functioning and health-related quality of life in older adults. This meta-analysis (*n* = 18 studies) found moderate positive effects of yoga (around *d* = 0.5) for a variety of physical effects as well as for depressive symptoms and quality of sleep, but no effects for cognition or attentional variables.

In contrast to the scarcity of studies on the impact of body-centered yoga techniques, there have been hundreds of studies on the impact of meditation, both for physiological and brain measures and psychological variables (see Murphy et al., 1997, for a large collection of early studies). The psychological effects of meditation have been summarized in several meta-analyses for nonclinical groups of practitioners, mostly conducted by members of the Maharishi International University (Eppley et al., 1989; Alexander et al., 1991, 1994a). These meta-analyses found strong effects on measures of trait anxiety and self-actualization, and strong reductions in drug use, as well as a general superiority of Transcendental Meditation, an approach that is commonly traced back to the ancient Advaita Vedanta system, compared to other methods. However, these analyses looked at only a small number of dependent variables (mostly trait anxiety and self-actualization) and there were several methodological problems connected with a substantial number of studies used in the analyses. Quite a few of the studies used only a pre–post design without a control group, which leads to low internal validity, and effect sizes in such studies tend to be overestimations (Dunlap et al., 1996).

According to Samkhya-Yoga (as well as other Hindu and Buddhist systems), the practice of meditation should lead to benefits on basically all psychological dimensions that can be conceived of in a positive–negative dimension. This was indeed the common result in a recent comprehensive meta-analysis (Sedlmeier et al., 2012) comprising 163 studies. This meta-analysis found an overall effect size (*d* = 0.58) comparable to that obtained in psychotherapy studies. The effects varied, however, for different types of variables. Effects were strongest—medium to large according to Cohen's (1992) conventions—for emotionality and relationship issues, and less strong (about medium) for variables that measured attention and cognitive measures. Due to the scarcity of studies that examined a given specific approach to meditation, they could be grouped into only three coarse categories: studies done with (a) Transcendental Meditation, (b) Buddhist meditation techniques, and (c) other techniques. Overall, and in contrast to the previous meta-analyses that found superior effects for Transcendental Meditation, there was no difference in global effect sizes for these three groups, although they differed in respect to several variables. Transcendental Meditation studies yielded comparatively large effects for the reduction of anxiety and negative emotions, and for learning and memory; Buddhist meditation techniques showed higher effects for the reduction of negative personality traits, stress reduction, and the improvement of attention and mindfulness than the other two categories; and even the “other” category had a comparatively strong effect in measures of cognitive ability. There are even pronounced differences in effects within specific categories of meditation such as the Buddhist meditation techniques: Whereas “pure” meditation had the highest effects on mindfulness and attention, additional breathing exercises and body postures led to stronger effects for most other psychological variables examined in the respective studies (Eberth and Sedlmeier, 2012).

The results reported by Sedlmeier et al. (2012) were stable according to several bias analyses, and in contrast to the clinical studies reviewed by Goyal et al. (2014), meditation remained superior to methods used by active control groups, such as groups receiving cognitive restructuring or relaxation training. Thus, one might regard the empirical effects of meditation in healthy populations as more or less established. However, as yet there is little evidence about the impact of the context of meditation.

#### Further research

In sum, there is indeed sound empirical evidence in favor of the cognitive training hypothesis, based on examining the impact of practicing the eightfold path to some degree. There is some indication that effects might be stronger if the yoga path is applied in a more comprehensive manner, combining both body-centered and meditation exercises. However, some more specific aspects of the cognitive training hypothesis have, to the best of our knowledge, not been examined, so far.

One of these aspects concerns the expected effects and their measurement. In the preceding paragraph, we already mentioned that according to Samkhya-Yoga, effects that can be described as a preponderance of the sattva guna should increase with increase in yoga practice. A corresponding decrease is to be expected for the five kleshas. We are not aware of any systematic attempt to construct a klesha questionnaire that would allow for measuring the effects of yoga practice. A central effect associated with diminishing kleshas is a decrease in the fluctuations of the mind; and a potential way to measure fluctuations of the mind might be to use the measurement devices developed in research on mind wandering. There are indeed first results that indicate a systematic effect of (mindfulness) meditation on mind wandering (e.g., Mrazek et al., 2013).

A second aspect not yet examined relates to the practice itself. The practice of yoga according to the Yogasutras consists of eight clearly specified stages, which have not been examined, so far, in their entirety. In addition to comparing the combined effect of the eight stages to effects of single stages (such as body or breathing exercises), it would also be very promising to examine the relative impact of the different stages of the path. To the best of our knowledge, the impact of the ethical part of yoga, and that of inner spiritual discipline have not been examined at all, so far. It is also still an open question whether meditation alone, that is, any of the four last stages of the eight-fold path or combinations thereof are sufficient to reach the postulated effects or whether the other four parts are a necessary requirement^8^.

A third interesting aspect that combines the two issues discussed above concerns person specific recommendations and might be examined in an exploratory manner, starting with an initial measurement of the “guna-mixture” for a given person. Dependent on meditators' personalities it might be especially beneficial if they concentrated on specific items of the eight-fold path or different combinations thereof (for a related approach see Frawley and Summerfiled Kozak, 2001). This is an issue dealt with in the traditional teacher student relationship in Hindu yoga practice. There, the teachers administer specific practices to their students dependent on the latters' personalities and progress on the path. So one way to start this exploratory research might be to interview experienced teachers (gurus).

### Extraordinary cognition hypothesis

When practicing the eightfold path described in the Yogasutras, one may acquire extraordinary forms of cognition, such as knowledge of the past and future, clairvoyance, clairaudience, psychokinesis, and telepathy (among others). According to the Yogasutras (verse 1 of book 4), such powers can be acquired by birth (as the result of accumulated karma), or from herbs, but they also come in a systematic way from yoga practice. To the best of our knowledge, the scriptures do not differentiate between the effects of specific items of the eight-fold path but one might argue, consistently with Buddhist assumptions, where one mainly expects the effects of extraordinary cognition to arise with concentrative techniques (Shamata meditation—see Buddhagosa, 2010) that the concentrative parts of yoga are especially suited to produce such extraordinary abilities (*siddhis*). Although the scriptures are not explicit about this, the main mechanism responsible for such abilities might be tied to the nonlocal nature of the three aspects of the mind as conceived in Samkhya-Yoga. There, (as well as in many other Indian systems), such extraordinary abilities are not seen as central, though. On the contrary, they are often regarded as hindrances to experiencing the world (including ourselves) as it really is (e.g., De Silva, 2005; Braud, 2008; see also verse 37 of the third book of Patañjali's Yogasutras; for an opposing contemporary view see Phillips, 2009, p. 62).

Because the achievements of previous lives (*karma*) also play an important role, one would expect some evidence for such *siddhis* in a randomly selected sample because any sample can be expected to contain people who either practice yoga now or have practiced it in their former lives. However, the general hypothesis would be that the more advanced yoga practitioners are (especially in respect to their concentrative meditation practice), the more likely extraordinary abilities are to arise. There are only a few studies that directly examined the extraordinary cognition hypothesis but its examination makes much more sense if one could show that the postulated effects exist at all. This more basic hypothesis has been researched for more than a century in Western psychology (e.g., James, 1898; Kelly et al., 2007) and we will first give a quick summary of the results. The topic has never made it into mainstream psychology although there is a long tradition of research on the respective phenomena, often termed *psi* effects, following a suggestion by Thouless and Wiesner (1947). As far as psi phenomena such as telepathy, clairvoyance, precognition, and psychokinesis are concerned, a minority of researchers established their connection to an Indian background a long time ago (e.g., James, 1896, 1898; Myers, 1903) and this was renewed very recently, but usually not by psychologists (e.g., Targ, 2012; Radin, 2013). Notable exceptions are researchers (including quite a few nonpsychologists) working on a revival of ancient Indian psychology (e.g., Rao et al., 2008; Rao, 2010; Cornelissen et al., 2014).

#### Summaries of psi research results

Research on psi phenomena (parapsychology and psychic phenomena are used as synonyms here) has produced a huge body of results that were collected largely in carefully controlled trials (e.g., Utts, 1991). However, almost all of these studies were *not* concerned with the impact of yoga practice or meditation, and quite often had undergraduates (and not advanced practitioners of yoga) as participants.

We cannot provide a comprehensive overview of all these research results, old and new, for want of space (see Radin, 2006, 2013; Rao, 2010, for such overviews, and May, 1995, 1996; Targ, 2012; Radin, 2013, p. 268, for some striking anecdotal examples), but we will summarize the results of research traditions within psi research that have produced enough studies to allow for meta-analyses. Table 1 gives an overview on the size of effects found in four areas, into which psi research is often divided. All the effect sizes given in Table 1 can (roughly) be interpreted as standard deviation units (*d*) even though the original effect sizes were sometimes expressed differently (for details see Honorton and Ferrari, 1989, p. 283; Utts, 1996, p. 24; Radin, 2005, p. 96).

**Table 1:** **Results in meta-analyses on psi phenomena, divided up into four areas of research: telepathy (receiving information from somebody distant in space), clairvoyance (perceiving events at a distance in space), precognition (receiving information about future events), and psychokinesis (influencing physical objects or physical and physiological processes by one's conscious intention)**.

**Area of psi research**	**Brief description of task**	***ES***	***N* (Studies)**	**Authors**
Telepathy	Ganzfeld effect^a^	0.20	11	Honorton et al., 1990
	same	0.14	29	Storm et al., 2010a^b^
Clairvoyance	Perceiving or “viewing” and describing an unknown randomly chosen remote geographical target to which an agent had been sent	0.20	1215	Utts, 1996
	same	0.21	653	Dunne and Jahn, 2003
Precognition	Predicting cards and numbers to be randomly chosen later on	0.02	309	Honorton and Ferrari, 1989
	Human physiological processes as a predictor of future important or arousing events	0.21	26	Mossbridge et al., 2012
Psychokinesis	Human intention on the output of random-number-generating device	0.003	832	Radin and Nelson, 1989
	same	0.01	148	Radin and Ferrari, 1991
	same	0.001^c^	377	Bösch et al., 2006
	Human intention on electrodermal activity (EDA) of people at a remote place [two meta-analyses]	0.11	36	Schmidt et al., 2004
		0.13	15	
	same	0.09	60	Radin, 2005
	Human intention on performance of a second remote person in an attention task at randomly chosen time intervals	0.11	11	Schmidt, 2012

*Effect sizes can be interpreted as standard deviation units and are rounded to two decimals unless the first two decimals are zero*.

a *In a typical Ganzfeld (German: “whole field”) experiment, a “receiver” is asked to relax in a comfortable chair in a sound-isolated, electrically shielded room. She wears halved Ping-Pong balls over her eyes and headphones that play pink noise and a lamp shines red light on her face—all procedures that are expected to make the receiver as receptive as possible. The receiver is asked to describe (into a microphone) the (randomly chosen) target material that is being observed and “sent” by a “sender” in a distant, equally prepared room*.

b *This meta-analysis also reviews several other meta-analyses on the ganzfeld effect, which on average yielded effect sizes comparable to the one in this study*.

c *Bösch et al. (2006, Table 4) report a mean proportion for all studies (here: their result for the random effects model without three outliers), which was transformed into a mean correlation using the formula they give in the note to their Table 1 (p. 499). This correlation was then transformed into standard deviation units (d) assuming equal sample sizes (see Rosenthal and Rosnow, 1991)*.

The mean effect sizes shown in Figure 1, are not higher than what is conventionally considered a small effect (*d* = 0.2, see Cohen, 1988) and sometimes even much smaller, especially for psychokinesis studies. Is this small (but stable) overall effect consistent with the predictions of Samkhya-Yoga (and other Indian systems)? We think that it could be interpreted in this way. One should keep in mind that Samkhya-Yoga mainly predicts the possibility of the arising of *siddhis* or extraordinary abilities for practitioners of yoga and meditation only after a period of intense practice, whereas studies conducted in psi research usually were done with inexperienced participants (inexperienced both in psi research and meditation), for whom one would not expect much of an effect.

Although the results of the psi studies reported here are consistent with the predictions of Samkhya-Yoga, this does of course, not exclude other explanations (for a recent brain-based model, see Marwaha and May, 2015).

#### Alternative explanations for psi effects

The history of psi research was and is accompanied by hot debate (e.g., Hyman, 1996, 2010; Storm et al., 2010b; Baptista and Derakhshani, 2014). Some researchers seem to take the existence of these phenomena for granted, whereas others a priori deny the possibility of such effects (e.g., Wiseman, 2011). A third group of scholars seems to be skeptical but open to both the possibility of such phenomena and different explanations for them (e.g., Schooler et al., 2011). Let us have a look at the main arguments that have been brought forward against the validity of psi effects. One argument is the difficulty of reproducing consistent results (e.g., Atmanspacher and Jahn, 2003; Kennedy, 2003), and another is that in the respective meta-analyses, outliers are removed and data are combined across inconsistent databases, therefore making the interpretation of average effect sizes arbitrary (e.g., Hyman, 2010). A possible counterargument, at least for the first point, would be that data of any kind are (and actually should be expected to be) inconsistent, that is, results from different studies should vary, because of the ubiquitous sampling error (Hunter and Schmidt, 1990). Alcock (2003) listed several other problems with psi research apart from methodological shortcomings: lack of a good theory and of a proper definition of the subject matter, problems with the definition of constructs, lack of progress, failure to jibe with other areas of science, and disinterest in competing hypotheses. However, none of these criticisms seems to be an insurmountable obstacle to serious scientific enquiry, and even skeptics admit that researchers on parapsychology have a high statistical and methodological sophistication (Hyman, 2010).

#### Further research

Overall, the data on psi experiments available, especially those summarized in meta-analyses (see Table 1), cannot be explained away as null results and definitely need some explanation. However, it also seems to us that, to date, the greatest deficit in psi research is the lack of a comprehensive theory. The Indian approach detailed above might be a starting point for such a theory although it also lacks detailed descriptions of the potential processes involved. Still, it advances an interesting and testable hypothesis not to be found in previous Western theorizing, for which there is also some confirming evidence: Psi phenomena should be much more prevalent with experienced practitioners of a yoga path than with arbitrarily chosen participants.

Tasks such as choosing the right number, card or picture out of several possible options, a kind of task often used in psi studies, are probably not what the originators of the ancient Indian theories had in mind when they spoke about *siddhis* or extraordinary cognitive abilities. In fact, in psi studies, larger effects were usually found for more complex stimuli (Radin, 2013). And there is indeed some indication that yoga practice might have an impact on cognitive abilities. In general, meditators do better than nonmeditators in studies in which this variable (meditation experience) has been taken into account (see Radin, 2013), and there are a few studies that explicitly examined this question and obtained markedly better results for experienced meditators (Schmeidler, 1970, 1994; Roney-Dougal and Solfvin, 2006, 2011; Roney-Dougal et al., 2008). The respective evidence is, however, still scarce and further research should focus on examining this hypothesis.

### Relative vs. absolute reality hypothesis

Samkyha-Yoga holds that there is a clear distinction between purusha, the absolute reality or pure consciousness (or true self), and the relative one, prakriti. Whereas prakriti, which also includes the different aspects of the mind, is material, purusha is non-material, stable, unchanging, and without qualities, that is, it could *not* be regarded as a trait in the conventional sense. This hypothesis in its general form also applies to all other Hindu systems of thought. According to all Hindu (and also Buddhist) schools, true reality can be perceived by people who have attained the ultimate goal of the respective yoga path, that is, enlightenment or liberation. There are plenty of first-person accounts of enlightenment experiences, especially of Eastern practitioners, and even more accounts of students of people who claim (or are claimed by their students) to have attained the state seen as the endpoint of the respective practice prescribed. However, all the available evidence consists of subjective reports (e.g., Bucke, 1961; Kapleau, 1989; but see Newberg et al., 2002).

Some readers might ask whether this is a hypothesis that can be scientifically examined at all. But one could argue that enlightenment and the associated postulated perception of absolute reality is just a construct. So it would, at least in principle, be comparable to other constructs in psychology such as intelligence, anxiety, or narcissism, to name a few, although arguably associated with a much lower baseline probability of existence. The basis for measuring established psychological constructs is nothing but some kind of verbal response that might eventually be formalized in the form of a questionnaire. So to us, there seems to be no convincing a priori argument why the relative vs. absolute reality hypothesis should not be open to empirical scrutiny. How could the ancient Indian sages possibly have come up with the hypothesis of a relative vs. an absolute reality? If our assumption holds that the theory contained in Samkhya-Yoga is fully empirically based, these sages must have had some experiences based on the practice of yoga that were far removed from usual experiences and not explainable by conventional knowledge. The Yogasutras and other texts also make it clear that having gained access to pure consciousness, which means to see this absolute reality, goes far beyond what can be expected according to the extraordinary cognition hypothesis.

For the moment, the possibility that the relative vs. absolute hypothesis is wrong cannot be excluded. The hypothesis might have initially resulted from a perceptual illusion due to sensory deprivation or perceptual isolation, which, for instance, seems to be a plausible explanation for some meditation induced light experiences (Lindahl et al., 2014). Later practitioners who knew about these reports might have had similar experiences solely due to expectancy effects. However, given the huge variety and number of enlightenment accounts, this possibility seems quite unlikely, and accepting it without further inquiry would cut off a potentially exciting line of research.

Therefore, it seems worthwhile to examine the experiences of practitioners who are seen as having reached a state of enlightenment more systematically, to get a clearer picture of what the access to absolute reality might be like. The main approach here has to be of a qualitative nature, but this does not exclude the necessity to also investigate the neurophysiological correlates of these experiences. Indeed, preliminary results with advanced practitioners of a Burmese Vipassana tradition seem quite promising (Davis and Vago, 2013). There have also been qualitative studies with very advanced practitioners from the same tradition, which hint at specific experiences that might be related to an experience of pure consciousness (Full et al., 2013).

If an absolute reality exists, its perception should not be different for practitioners of different traditions, although it might be expressed differently in these traditions. Thus, it might be worthwhile to collect already existing personal accounts that describe enlightenment experiences and check them for consistency^9^. But it might be even more worthwhile to conduct studies, maybe even a longitudinal ones, with long-term practitioners who could take over the roles of both researchers and participants. One could argue that meditation is not only a topic of research but can also serve as a research method. There is some evidence that the introspective accuracy of meditators increases with meditation (Vago and Nakamura, 2011; Fox et al., 2012; Van Vugt et al., 2012). Eventually, practitioners, once enlightened, may come to agree (or disagree) on a common account of what it means to have access to an absolute reality. Such an agreement might also mean that practitioners from some or perhaps even all traditions give up tenets from their tradition, thus helping arrive at an ever more precise version of the relative vs. absolute reality hypothesis. And even if it eventually turned out that the relative vs. absolute reality hypothesis was untenable, a careful examination of practice-induced changes in cognition and consciousness could be extremely valuable for advancing our understanding of mental processes in general.

The approach to be used in such designs has to be largely of a qualitative nature (see Gergen et al., 2015, for a recent motivation to use this kind of methodological approach more comprehensively). Especially suited seems to be a special kind of a qualitative approach, termed the “second person” method, where the second person must be experienced and knowledgeable about the topic in question (meditation or yoga), must be trained in helping the “first person” in his or her introspection, and must be able to develop good personal terms with the “first person” (e.g., Varela and Shear, 1999). Recently, phenomenological approaches have been suggested that might be well suited for examining practitioners' understanding of changes in consciousness and cognition that are not open to conventional measurement (Lutz and Thompson, 2003; Thompson, 2009; Zahavi, 2010).

Such a collaborative research endeavor, if done open mindedly, would have several advantages compared to the traditional researcher–participant setup. Obviously, the qualitative approach is restricted to mental processes we have access to, but this access is potentially far better than usually assumed (Petitmengin-Peugeot, 1999; Petitmengin, 2006; Petitmengin et al., 2013).

## Future theory and research

It seems fair to say that, taken together, there is considerable empirical evidence for at least some of the diverging hypotheses derived from Samkhya-Yoga and that some of these hypotheses, if made more precise, have the potential to enrich existing psychological theories. There are, however, still many gaps that have to be filled, both in theorizing and research methods.

### Issues of theory

Above, we have dealt with Samkhya-Yoga and Indian systems in general as if they were “ordinary” psychological theories or hypotheses. Some researchers involved in the Indian psychology movement (e.g., some contributors in Rao et al., 2008; Cornelissen et al., 2014) might not totally agree with this. However, we conceive of these systems as theories that were built to explain ordinary experiences but also insights exceptionally gifted and well-trained people had in ancient India, and therefore, we think these theories should be open to empirical scrutiny without any restrictions (see also Sedlmeier et al., 2014). Our suggestion, however, is not to take the Indian systems as alternatives to Western cognitive psychology. The Indian system dealt with here (and others, such as early Buddhism, or Advaida Vedanta) are much broader in scope than a randomly selected theory in Western psychology and comprise basically all psychology-relevant aspects that were known at their time of origin. However, they are certainly incomplete in many respects and their embedment in religious and philosophical contexts might also turn out to be a restriction in some ways. For instance, the concept of karma, that is, the idea that previous actions (including those performed in previous lives) have an effect on present lives might be hard to even provisionally accept by contemporary Western scholars. However, if one only looks at the postulated effects, one might, for pragmatic reasons, want to think of the karmic heritage from previous lives as genetic factors (that also stem from previous lives, that is, the lives of our ancestors) and one could conceive of the effects of previous deeds as being part of our implicit and explicit memories.

The contents of the Indian systems were presumably derived by direct experience, which might have yielded insights that are not possible to gain in the way Western psychology has been mostly practiced. These insights and the corresponding research hypotheses are potentially relevant for Western psychology, but existing research methodology may come to its limits in examining them empirically.

#### Related western theoretical approaches

To the best of our knowledge, as yet, there does not exist a Western comprehensive theory of the effects of yoga (understood in a broad sense), but there have been several attempts to explain the effects of meditation, mainly drawing on Buddhism and incorporating Western theoretical concepts. Whereas some of the theoretical explanations made close connections to specific Buddhist systems (e.g., Lutz et al., 2007; Grabovac et al., 2011), most accounts focused mainly on the concept of (secular) mindfulness and attempted to explain the effects of mindfulness practice. Postulated mechanisms include “metacognitive awareness” (Teasdale et al., 2002; Vago and Silbersweig, 2012), “positive reappraisal” (Garland et al., 2009), changes in intention, attention, and attitude (Shapiro et al., 2006; Lutz et al., 2008), or a combination of processes such as attention regulation, body awareness, emotion regulation, and change in perspective on the self (Hölzel et al., 2011; Teper et al., 2013). It seems very promising to make these previous approaches more comprehensive by incorporating more of the theoretical background that already exists in the Indian systems (see also Awasthi, 2013), where meditation is not some arbitrary collection of techniques that might be used for some beneficial health-related purposes but is a necessity to achieve the highest form of cognition in the respective systems. Having this background in mind, key issues in meditation research might, as already mentioned, be the relative importance of parts of the yoga path, such as moral aspects or aspects that concern the conduct of daily life. Moreover, an important issue in this research might be the role of the religious background. Is it, for instance, necessary to believe in a personal god (as suggested in the Yoga but not the Samkhya system) to obtain the full benefits of yoga practice? A comprehensive theory of meditation does not necessarily have to include all the processes postulated in a given Indian approach but the mechanisms postulated there can be a good basis on which to build such a theory and to derive empirically testable predictions.

#### Mind–brain relationship

Especially interesting for future theorizing might be the discrepancy in views about the mind–brain relationship between the Indian approaches and the mainstream Western view. For most Western researchers there is no doubt that all cognitive processes including consciousness are produced by the brain (e.g., Ainslie, 2001; Dennett, 2004; Damasio, 2012). This view is immediately evident in the computational theories of the mind and especially in connectionist theories, but even critics of such theories regard it as an axiom that cannot be doubted (e.g., Searle, 2000). The view is well illustrated by a quote from a talk by Antonio Damasio at the concluding ceremonies of the 1999 “Decade of the Brain” project:

In an effort that continues to gain momentum, virtually all the functions studied in traditional psychology—perception, learning and memory, language, emotion, decision-making, creativity—are being understood in terms of their brain underpinnings. The mysteries behind many of these functions are being solved, one by one, and it is now apparent that even consciousness, the towering problem in the field, is likely to be elucidated before too long” (as cited in Kelly, 2007, p. xx).

However, how this should work is still something of a mystery, expressed in Chalmers's (1995) *hard problem of consciousness*: “Why should physical processing give rise to a rich inner life at all? It seems objectively unreasonable that it should, and yet it does” (p. 201). How does consciousness arise out of the functioning of the human brain and how is it related to the behavior that it accompanies?

The Indian systems obviously did not directly deal with the brain because some 2000 years ago, there was no way to monitor brain activity. But brain definitely consists of gross matter, whereas consciousness in the system of Samkhya-Yoga is clearly defined as being nonmaterial. In general, Indian systems regard the brain as an instrument used by the mind (e.g., Raju, 1983). That the mind is assumed to go beyond the brain is also consistent with the hypothesis of the extraordinary abilities (*siddhis*) postulated by all major Indian systems.

Interestingly, there has always been a small minority in Western academic psychology that did not accept the view that consciousness is merely an epiphenomenon of brain activity. James stated as early as 1899 that “matter is not that which *produces* Consciousness, but that which *limits* it, and confines its intensity within certain limits” (James, 1899; p. 67). A summary of views and empirical results of researchers adhering to this minority view is given in Kelly et al. (2007). The findings summarized above might be taken as a motivation to further explore the issue of the mind–brain relationship without a priori excluding the possibility of non-brain based consciousness.

### Issues of research methods: Study design and measurement

Research on the impact of yoga and meditation on cognition can certainly be done using conventional study designs and with the methodological tools already available in Western psychology, but confining oneself to established ways and tools would limit the range of research questions that can be examined. For instance, to examine more specialized questions about the effects of yoga, it might be hard to find large homogeneous samples of participants. Rather than seeing this as a hindrance, one could take it as a chance to have a closer look at every individual, thus allowing for more detailed measurement and also allowing for the measurement of processes (instead of only results).

Apart from the second-person methods mentioned above, using a qualitative approach, so-called *single-case experimental designs* that allow for drawing causal inferences in a way similar to randomized group designs might be very useful in examining hypotheses derived from the Indian systems. Instead of randomizing over people (as in group designs), these designs, such as *multiple-baseline* or *alternating treatment* designs, use randomization over time to control for causal influences other than the independent variables in question (see Barlow et al., 2009; Sedlmeier et al., 2016).

Probably due to the largely atheoretical way in which meditation research has, until recently been done, effects of meditation have been measured in basically every conceivable way (Sedlmeier et al., 2012). If, however, measurement is based on theory, as it should be, Samkhya-Yoga would, for instance, suggest measuring changes in the three gunas, and in the kleshas. We believe that to examine the hypotheses advanced above, it is necessary to rethink approaches to measurement, and it will also be necessary to expand existing methodologies or develop new ones (Sedlmeier et al., 2016).

## Conclusion

In this article we claim that ancient Indian theories of cognition have indeed something to offer mainstream Western psychology. While large portions of the Indian theories are mirrored in contemporary Western conceptions of cognition, there *is* something new in the predictions that can be derived from these systems. The ancient Indian systems contain some hypotheses that are not part of mainstream Western academic psychology and they provide starting points for theoretical explanations for phenomena that have been researched in the West, albeit without a sound theoretical basis. They provide some new hypotheses about means to improve one's life and the expected outcomes thereof and can be taken as a basis for a comprehensive theory of meditation. Moreover, they propose a different or extended view of reality by postulating a higher form of consciousness rarely dealt with, so far, in Western psychology.

Such a research endeavor is not possible, however, if there are a priori restrictions in the range of possible theoretical assumptions, for example, about the nature of consciousness. Although it might be hard for many Western psychologists to even consider the possibility of something like the “absolute reality” or the extraordinary forms of cognition discussed above, scientific psychology could lose much if these hypotheses are not given a good chance to be examined empirically and put to the test. If the results eventually turn out to be inconsistent with the hypotheses advanced by the Indian systems, having empirical justification for this conclusion would be vastly better than just believing a priori that the respective hypotheses are invalid. If, on the other hand, some of the hypotheses introduced by the Indian systems turned out to withstand rigorous scientific scrutiny this could yield an enormous enrichment of our current psychological theorizing.

## Author contributions

All authors listed, have made substantial, direct and intellectual contribution to the work, and approved it for publication.

### Conflict of interest statement

The authors declare that the research was conducted in the absence of any commercial or financial relationships that could be construed as a potential conflict of interest.

## Footnotes

^1^One might argue that another later system, Advaita Vedanta, is the most famous Indian system, and its greatest authority, Shankara, might be the most famous Indian philosopher-sage ever to have lived (Flood, 2004, p. 239), but the theory of cognition in Advaita Vedanta was very much influenced by Samkhya-Yoga and is similar to it in many respects. Therefore, and also for want of space, we omitted Advaita Vedanta in this paper. The system of (early) Buddhism (based on the so called *Pali Canon*) would also have been a promising candidate but we decided against it for want of space. Other Hindu systems such as Nyaya and Vaisheshika are in many respects very similar to Western thinking and therefore would not add much to the main argument brought forward in this paper.

^2^There is some disagreement as to when, exactly, the Vedas (there are four, consisting of four parts each) were written: Estimates for the earliest Veda (the Rig Veda Samhita) range between 3500 BCE and 1200 BCE (e.g., Hiriyanna, 1932/2000, Sharma, 1960/2003, Frawley, 2001; Klostermaier, 2006).

^3^Most scholars make a distinction between the so-called orthodox systems, such as Samkhya-Yoga that are directly based on the Vedas or reactions to them, the so-called heterodox systems, such as Buddhism. Apart from Samkhya and Yoga, there are four other orthodox systems that are also usually treated as pairs: Nyaya and Vaisheshika, and Mimamsa and Vedanta. Advaita Vedanta is a variant of the system of Vedanta. Besides Buddhism, other important heterodox systems are Carvaka, a materialistic approach, and Jainism. Although traditionally, most experts in the field seem to agree with this distinction, some scholars argue that the difference between orthodox (astika) and heterodox (nastika) schools might have been exaggerated, Buddhism and Jainism having also belonged to the “orthodox” class at some time (Nicholson, 2010). A case in point would also be that later (intermediary) Buddhist developments came very close to Hindu systems of thought: For instance, the Sarvastivada and Sautantrika schools of Buddhism had much in common with the Samkhya system (described below in some detail), and the Vaisheshika school (Kalupahana, 1991, p. 22).

^4^In Samkhya-Yoga and basically all other Indian theoretical approaches, the different aspects of mind are seen as material. However, the material parts are so subtle that they, for instance, cannot be seen by normal vision (similar, one could argue, to the *Chi* postulated in Chinese medicine). The difference in subtleness assumed by the theory is probably due to the different functions of the three aspects of the mind. The sense mind is directly connected with the material world (and therefore less subtle) whereas the intellectual mind is responsible for the connection to the non-material purusha (and therefore most subtle).

^5^In the classic account, the yogi who has achieved liberation, that is, dwells in pure consciousness, gives up contact with mundane life because staying there will destroy his knowledge and bring him back to a life of bondage (e.g., Jayashanmugam, 2002). This assumption has, however, been questioned by more recent yoga philosophers (“yoga” in small letters standing here for the practice, in contrast to the Yoga system) such as Swami Vivekananda and Rama Tirtha (Srinivas, 2004); and also Sri Aurobindo's Integral Yoga claims that the goal of liberation can be reached within everyday life (Sri Aurobindo, 1996; Maitra, 2001). A similar view seems to be held by contemporary authors (e.g., Phillips, 2009).

^6^The term *yoga* stands for a collection of different practices and is not to be mixed up with the philosophical system of *Yoga* (here written with a capital *Y*). Apart from the approach described above, there are many more (for an overview, see (Feuerstein, 2001); see also (Phillips, 2009), for more recent developments). Here we just mention some of these alternative approaches, which all relate in some way to the Yogasutras and which all have the aim of liberating the practitioner from a limited experience of the world. Three other main approaches already described in the Bhagavadgita are jnana yoga, bhakti yoga, and karma yoga. Jnana yoga, the path of knowledge, relies heavily on the study of old scriptures and on intellectual insight; bhakti yoga, the path of devotion, cultivates the practice of devoting one's life to the love of God; and karma yoga, the path of action, consists of doing everything without being attached to the fruits of action. In Kundalini yoga, practitioners work to awake Kundalini, some kind of spiritual energy, and in Mantra yoga, (holy) syllables or words (such as OM) or phrases are used (usually internally repeated) for the purpose of meditation. There are also somewhat paradoxical kinds of yoga, usually summarized under the name of tantra yoga, in which rules stuck to in the conventional yoga systems such as eating no meat or abstaining from sexual activity are systematically broken to free the mind from the (relative) perceptions of the world and enable the practitioner to gain insight and liberation.

^7^Whereas hedonism refers to the usual conception of well-being, that is, striving for maximization of pleasure (positive affect) and minimization of pain (negative affect), proponents of the concept of eudaemonia (such as Aristotle) hold that true happiness is found by leading a virtuous life and doing what is worth doing, even if doing so might not be immediately pleasing or rewarding.

^8^Similar issues are currently discussed for secular mindfulness-meditation programs that originally stem from Buddhist approaches (e.g., Amaro, 2015).

^9^Here, there is the obvious problem of how to tell whether somebody has attained enlightenment. In some forms of yoga (understood in a broad sense), for instance, in Zen, the respective diagnosis is made by the teacher who is also supposed to be enlightened, but even there some space for doubt and uncertainty remains (e.g., Coleman, 2001; Samy, 2013, Chapter 8).
