# Supplementary material for: How Do Theories of Cognition and Consciousness in Ancient Indian Thought Systems Relate to Current Western Theorizing and Research?
Source: Front Psychol. 2016 Mar 15;7:343. doi: 10.3389/fpsyg.2016.00343 (PMC4791389; doi:10.3389/fpsyg.2016.00343)
Supplement: Supplementary file 1 [file Table2.pdf]

Glossary of Sanskrit Terms Used in the Article

Attachment to or involvement in the thirst, greed, and craving for enjoyment	*raga*
Body postures	*asana*
Breath control	*pranayama*
Cognition (single unit of awareness)	(*citta*) *vritti*
Concentration	*dharana*
Contemplation	*dhyana*
Control of the senses	*pratyahara*
Desire to continue to be what one is and the instinctive fear of death	*abhinivesha*
Ego mind	*ahamkhara*
Energy	*rajas* (*guna*)
Ethics	*yama*
Feeling of a (mistaken) personal identity	*asmita*
Hate or aversion of anything considered painful	*dvesha*
Hindrances	*kleshas*
Inertia	*tamas* (*guna*).
Inner spiritual discipline	*niyama*
Intellectual mind	*buddhi*
Meditative state of absorption	*samadhi*
Memories	*samskaras, vasanas*
Mind	*citta*
Nature	*prakriti* (consisting of the three *gunas*)

Purity	*sattva (guna)*
Sense mind	*manas*
Spiritual ignorance	*avidya*
